# The Present State of Panel Medical Practice

**Published:** 1913-10-25

**Authors:** 


### THE EDITOR'S LETTER-BOX.

### The Present State of Panel Medical Practice.

*To the Editor of* The Hospital.

Sir,—The Fabian Society has just announced its intention of instituting an inquiry into the actual working to date of the National Insurance Act. It would have been better, perhaps, especially as regards that part of it now under notice, to have waited a little longer, until, say, next April or May; because winter is by far the busiest time of year in a medical practice, and the response of the system to the test of a whole winter season would then have been available. However, certain noticeable phenomena have already become apparent. We will take, for instance, the case of a northern city of considerable size, with a dense industrial population also in the environs. Here, although at first the percentage of medical men going on the panel was large, now only 80 out of 200 odd have their names there. The explanation of this is that men with well-established practices of the kind known as "mixed" have resigned rather largely this last summer, their motive being probably the fear of losing—the anticipation even of multiplying—their socially better-class patients. There is no doubt that "medical benefit," the arrangement, that is, of paying doctors by contract instead of when actually required, has increased, at any rate for the present, the number of visits paid to, and paid by, them very much. That rather bare apartment, misnamed the "surgery," is, in a practice with panel patients, crowded out with the poorer members of society, and their presence has, as a matter of blunt fact, an inhibiting effect upon richer ones, who gravitate to where the company is more select. Besides, the fact of a medical man tacitly proclaiming himself independent of contract practice by not going on, or by leaving, the panel, undoubtedly increases his prestige, and incidentally relieves him of much clerical work, while he gains the satisfaction of feeling that his time is being paid for at a much better rate. For all these reasons panel practice (in districts such as those under notice) is at present tending to be carried on by those whose only private patients are, practically, the dependants of the panel patients themselves.

There are many fewer practitioners than at first seemed likely, then, to do panel work. Now, what does this mean? Obviously, that each panel doctor must have a great many clients—one can hardly call them patients. We have heard of a case where a young man of strong constitution has undertaken single-handed to look after 4,000. Another practitioner has even more, and the shifts whereby he manages somehow to get through with the job are, though not positively illicit, nevertheless highly undesirable. A relative who is a medical student makes himself generally useful. A qualified midwifery nurse, paid a large salary, undertakes the obstetric department, summoning her chief just before actual parturition, and doing all the visiting of the case afterwards. Books of prescriptions, ready written out for various regional ailments, are kept, the leaves being torn out and given to patients almost before they have done telling their symptoms. Surgical cases are shunted to the hospital. The expense of a large motor-car, such as those used by leaders of the profession, is easily met out of all this drug-selling, and makes possible the daily scurry round forty or fifty home cases.

The remedy that those with real opportunities of observation are inclined to favour is that panel practice must be spread out over a greater number of medical men; and, since the entry of medical students each year remains comparatively small, this means that it must be made compulsory. Every general practitioner should be bound to undertake so many panel patients. Here we have a State Medical Service at once—a tentative conclusion our Fabian friends will be far from quarrelling with. And while disclaiming any intention of prophesying them pleasant things, we do not at once see to what other destination the facts of the case lead one's judgment so directly. The insertion of the thin end of the wedge, or the first downward step, or the first true advance (whichever metaphor you please) took place when the stamps were placed on the first insurance cards. The widening, or the glissade, or the progress, began with the doctors' collapse last January. It would take something very revolutionary indeed to constitute a real new departure now.—I am, Sir, yours faithfully,

Northerner.

